# Deep Ensemble Model for COVID-19 Diagnosis and Classification Using Chest CT Images

**DOI:** 10.3390/biology11010043

**Published:** 2021-12-29

**Authors:** Mahmoud Ragab, Khalid Eljaaly, Nabil A. Alhakamy, Hani A. Alhadrami, Adel A. Bahaddad, Sayed M. Abo-Dahab, Eied M. Khalil

**Affiliations:** 1 Information Technology Department, Faculty of Computing and Information Technology, King Abdulaziz University, Jeddah 21589, Saudi Arabia; 2 Centre of Artificial Intelligence for Precision Medicines, King Abdulaziz University, Jeddah 21589, Saudi Arabia; 3 Department of Pharmacy Practice, Faculty of Pharmacy, King Abdulaziz University, Jeddah 21589, Saudi Arabia; keljaaly@kau.edu.sa; 4 Department of Pharmaceutics, Faculty of Pharmacy, King Abdulaziz University, Jeddah 21589, Saudi Arabia; nalhakamy@kau.edu.sa; 5 Center of Excellence for Drug Research and Pharmaceutical Industries, King Abdulaziz University, Jeddah 21589, Saudi Arabia; 6 Mohamed Saeed Tamer Chair for Pharmaceutical Industries, King Abdulaziz University, Jeddah 21589, Saudi Arabia; 7 Department of Medical Laboratory Technology, Faculty of Applied Medical Sciences, King Abdulaziz University, Jeddah 21589, Saudi Arabia; hanialhadrami@kau.edu.sa; 8 Molecular Diagnostic Lab, King Abdulaziz University Hospital, King Abdulaziz University, Jeddah 21589, Saudi Arabia; 9 Special Infectious Agent Unit, King Fahd Medical Research Center, King Abdulaziz University, Jeddah 21589, Saudi Arabia; 10 Information Systems Department, Faculty of Computing and Information Technology, King Abdulaziz University, Jeddah 21589, Saudi Arabia; dbabahaddad10@kau.edu.sa; 11 Mathematics Department, Faculty of Science, South Valley University, Qena 83523, Egypt; sdahb@fci.luxor.edu.eg; 12 Department of Mathematics, Faculty of Science, Al-Azhar University, Cairo 11884, Egypt; eiedkhalil@tu.edu.sa; 13 Department of Mathematics, College of Science, Taif University, Taif 21944, Saudi Arabia

**Keywords:** COVID-19, deep learning, ensemble models, machine learning, metaheuristics

## Abstract

**Simple Summary:**

Coronavirus disease 2019 is a worldwide pandemic posing significant health risks. Medical imaging tools can be considered as a supporting diagnostic testing method for coronavirus disease since it uses available medical technologies and clinical findings. The classification of coronavirus disease using computed tomography chest images necessitates massive data collection and innovative artificial intelligence-based models. In this study, we explored the significant application of computer vision and an ensemble of deep learning models for automated coronavirus disease detection. In order to show the better performance of the proposed model over the recently developed deep learning models, an extensive comparative analysis is made, and the obtained results exhibit the superior performance of the proposed model on benchmark test images. Therefore, the proposed model has the potential as an automated, accurate, and rapid tool for supporting the detection and classification process of coronavirus disease.

**Abstract:**

Coronavirus disease 2019 (COVID-19) has spread worldwide, and medicinal resources have become inadequate in several regions. Computed tomography (CT) scans are capable of achieving precise and rapid COVID-19 diagnosis compared to the RT-PCR test. At the same time, artificial intelligence (AI) techniques, including machine learning (ML) and deep learning (DL), find it useful to design COVID-19 diagnoses using chest CT scans. In this aspect, this study concentrates on the design of an artificial intelligence-based ensemble model for the detection and classification (AIEM-DC) of COVID-19. The AIEM-DC technique aims to accurately detect and classify the COVID-19 using an ensemble of DL models. In addition, Gaussian filtering (GF)-based preprocessing technique is applied for the removal of noise and improve image quality. Moreover, a shark optimization algorithm (SOA) with an ensemble of DL models, namely recurrent neural networks (RNN), long short-term memory (LSTM), and gated recurrent unit (GRU), is employed for feature extraction. Furthermore, an improved bat algorithm with a multiclass support vector machine (IBA-MSVM) model is applied for the classification of CT scans. The design of the ensemble model with optimal parameter tuning of the MSVM model for COVID-19 classification shows the novelty of the work. The effectiveness of the AIEM-DC technique take place on benchmark CT image data set, and the results reported the promising classification performance of the AIEM-DC technique over the recent state-of-the-art approaches.

## 1. Introduction

In December 2019, a new coronavirus disease 2019 (COVID-19) appeared in Wuhan, China, and has become a global healthcare emergency rapidly [1]. Because of its optimized medical resource assignment, higher infection rates and fast diagnoses in pandemic regions are crucial. Fast and accurate diagnoses of COVID-19 assist in isolating diseased persons could slow the disease spread. However, in pandemic regions, inadequate healthcare resource has become a major problem [2]. Hence, finding higher-risk persons with the worst prognoses for earlier special care and medical resource is critical in COVID-19 treatments. Now, reverse transcription (RT)-PCR is employed as a gold truth for the diagnosis of COVID-19. However, the shortage of testing kits and the limited sensitivity of RT-PCR in pandemic areas increases the burden of screening, and some diseased peoples are not isolated instantly [3]. This accelerates the spread of COVID-19. In contrast, owing to the absence of healthcare resources, some deceased persons could not receive prompt treatments. In such situations, detecting higher-risk patients with the worst prognoses for earlier prevention and treatments are significant. Subsequently, faster diagnoses and detecting higher-risk patients with the worst prognoses are highly useful for the management and control of COVID-19.

In order to alleviate the shortage and inefficiency of the present test for COVID-19, a large number of measures have been dedicated to searching for other testing systems [4]. Various researches using statistical models, machine learning (ML), and deep learning (DL) models have demonstrated that computed tomography (CT) scan manifests clear radiological findings of COVID-19 patients and serves as a more accessible and efficient testing method because of the wide accessibility of CT devices that could rapidly achieve results [5,6,7]. Furthermore, to mitigate the burden of healthcare experts in reading CT scans, several studies have proposed a DL method that could interpret CT images automatically and forecast whether the CT is positive for COVID-19 [5]. When this work has demonstrated a possible result, they have two constraints. Initially, the CT scans data set employed in this work is not shareable to the public because of privacy concerns. Subsequently, the CT images cannot be used by other trained models to diagnose the COVID-19 [6]. In addition, the limited availability of open-source annotated COVID-19 CT data sets considerably hinders the development and research of more innovative AI methods for precise CT-based testing of COVID-19. Next, this work requires a huge amount of CTs at the time model training to attain performances that meet the medical standards. These requirements are severe in practice and may not be confronted by several hospitals, particularly in the circumstance where healthcare experts are occupied highly by taking care of COVID-19 persons and are not likely to have time to annotate and collect huge amounts of COVID-19 CT scans.

The DL method as artificial intelligence (AI) method has demonstrated a possible result in assisting lung disease analyses through CT images [7]. Benefit from the stronger feature learning capability, DL could mine feature that is associated with medical outcome from CT image manually. Feature learned through DL methods could reflect higher dimension abstract mapping that is complicated for humans to sense; however, they are highly related to the medical outcome [8]. The transfer learning (TL) method aims to leverage data-rich source tasks to assist the learning of data-deficient targeted tasks (CT-based diagnoses of COVID-19). One frequently employed approach is to learn a strong visual feature extraction deep networks by pre-training this network on a huge data set in the source task and later adopt these pretrained networks to the targeted tasks by fine-tuning the network’s weight on the small size data sets in the targeted tasks [9]. In general, the TL model might be sub-optimal because the source data might contain a huge discrepancy with the targeted data based on the visual appearances of class labels and images that cause the feature extraction networks biased to the source information and generalize worse on the targeted information.

This study proposes an artificial intelligence-based ensemble model for the detection and classification (AIEM-DC) of COVID-19. Primarily, the Gaussian filtering (GF)-based preprocessing technique is applied for the removal of noise and improving image quality. Moreover, shark optimization algorithm (SOA) with an ensemble of DL models, namely recurrent neural networks (RNN), long short-term memory (LSTM), and gated recurrent unit (GRU), is employed for feature extraction. In addition, an improved bat algorithm with a multiclass support vector machine (IBA-MSVM) model is used as a classifier. The experimental validation of the AIEM-DC technique is validated on the benchmark CT image data set, and the results reported the promising classification performance of the AIEM-DC technique over the recent state-of-the-art approaches.

## 2. Literature Review

This section offers a brief review of existing COVID-19 diagnosis and classification models. Serte and Demirel [10] proposed an AI method for classifying COVID-19 and standard CT volume. The presented model employs the ResNet-50 DL models for predicting COVID-19 on all CT images of three-dimensional CT scans. Next, these AI methods fuse image-level prediction to detect COVID-19 on three-dimensional CT volumes. In Li et al. [11], an AI scheme has been proposed to manually quantify and segment the COVID-19 diseased lung region on a thick section chest CT image. The 531 CT images from 204 COVID-19 persons have been gathered from selected COVID-19 hospitals. The manually segmented lung abnormalities have been related to the automatic segmentation of two skillful radiotherapists with the Dice coefficients on arbitrarily elected subsets (30 CT scans). The two imaging bio-markers have been computed manually such as POI, and the iHU, for assessing diseases progression and severity.

Alshazly et al. [12] explore how a deep learning model trained on chest CT images could detect COVID-19 diseased persons in an automated and fast manner. Then, they adapted deep network architecture and presented a TL approach with customized input tailored to all the deep architectures for achieving better results. Yousefzadeh et al. [13] present ai-corona, a radiotherapist assistant DL model for COVID-19 disease diagnoses with chest CT scan. This model incorporates an effective NetB3-based feature extractor. They used three data sets: the CC-CCII set, MosMedData, and MDH cohorts. General, this data set constitutes 7184 scans from 5693 subjects and includes the normal class, COVID-19NCA, CP, and non-pneumonia. Hasan et al. [14] introduce the integration of DL models of extracted features using the Q-deformed entropy hand-crafted feature to discriminate among healthier CT lung images, COVID-19 coronavirus, and pneumonia. In this work, preprocessing is employed for reducing the effects of intensity differences among CT slices. Next, histogram thresholding is employed for isolating the background of CT lung scans. All the CT lung scans undergo a feature extraction that involves Q-deformed entropy and DL algorithms. The attained feature is categorized into an LSTMNN classifier.

In Shah et al. [15], the DL methods employed in the presented model is depending on CNN models. These manuscripts focus on distinguishing the CT scans of COVID-19 and non-COVID-19 CT images with distinct DL methods. A self-developed model called CTnet-10 has been developed for the COVID-19 diagnoses, has 82.1% of accuracy. Moreover, another methods that verified are VGG-16, DenseNet-169, VGG-19, ResNet-50, and InceptionV3. In Zheng et al. [16], a weakly supervised DL-based software framework has been proposed by three-dimensional CT volumes for detecting COVID-19. For all the patients, the lung regions are divided into a pretrained UNet; next, the separated three-dimensional lung regions were fed to a three-dimensional DNN for predicting the likelihood of COVID-19 disease.

Shalbaf and Vafaeezadeh [17] introduce an automated method that depends on an ensemble of deep TL for the diagnosis of COVID-19. The overall of 15 pretrained CNNs architecture: NasNetLarge, EfficientNet (B0-B5), InceptionV3, NasNetMobile, SeResnet 50, ResNet-50Xception, Inception_resnet_v2, and ResNext50 DenseNet121 are employed and later finetuned on the targeted tasks. Next, constructed an ensemble model according to the majority voting of optimal combinations of deep TL output for additionally improving the detection accuracy. Wu et al. [18] present a weakly supervised deep active learning model named COVID-AL for diagnosing COVID-19 by CT scan and patient-level label. The COVID-AL includes the lung region segmentation using two-dimensional UNet and the diagnoses of COVID-19 using a new hybrid active learning method that concurrently considers predicted loss and samples diversity.

## 3. The Proposed Model

**Figure 1:**
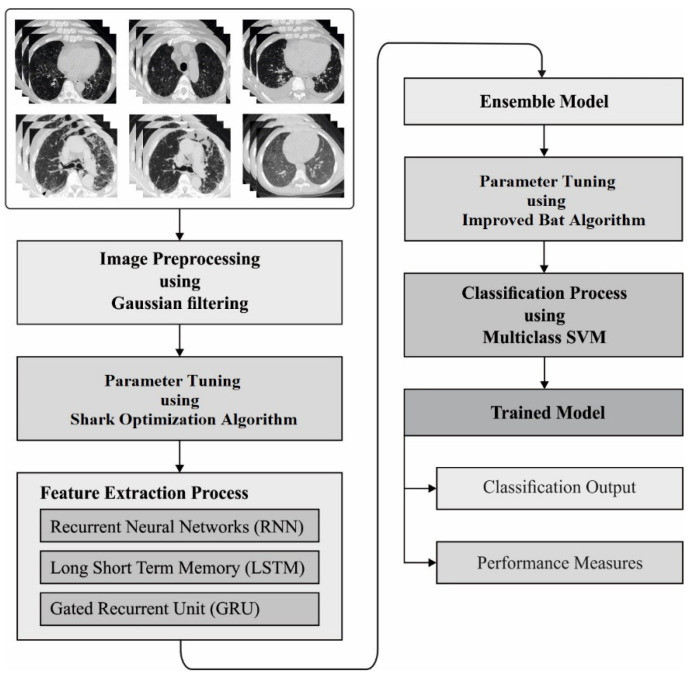
The process flow of proposed model.

In this study, a new AIEM-DC technique is proposed for the detection and classification of COVID-19 using chest CT scans. The AIEM-DC technique aims to accurately detect and classify the COVID-19 using an ensemble of DL models. The AIEM-DC technique involves GF-based preprocessing, ensemble DL-based feature extraction, SOA-based hyperparameter tuning, MSVM-based classification, and IBA-based parameter tuning. Figure 1 demonstrates the overall block diagram of the AIEM-DC model. These processes are elaborated in the succeeding sections.

### 3.1. Stage 1: Gaussian Filtering (GF)-Based Preprocessing

At the initial stage, the GF technique is applied for image preprocessing to eradicate the noise and boost the quality of the CT scans. The two dimensions GF has been used widely for noise elimination and smoothing. It requires huge processing resources and the efficacy in executing is a stimulating study. Convolution’s operator is determined as Gaussian operator, and suggestion of Gaussian smoothing is accomplished using a convolution. The Gaussian operators in *1D* are given below:

(1) $$G_{1D}\left(x\right)=\frac{1}{\sqrt{2\pi }\sigma }e^{-\left(\frac{x^{2}}{2\sigma^{2}}\right)}.\;$$

The optimal smoothing filters for an image undergo localization in the frequency and spatial domain, in which the ambiguity relations are fulfilled by [19]:

(2) $$\Delta x\Delta \omega \geq \frac{1}{2}.\;$$

The Gaussian operator in *2D* is demonstrated as:

(3) $$G_{2D}\left(x,y\right)=\frac{1}{2\pi \sigma^{2}}e^{-\left(\frac{x^{2}+y^{2}}{2\sigma^{2}}\right)},\;$$

whereas σ (sigma) represents the standard deviation (SD) of the Gaussian operator. While it contains a maximal value, the image smoothing will be high. (x, y) represent the Cartesian coordinate points of the image.

### 3.2. Stage 2: Ensemble Feature Extraction

During feature extraction, the preprocessed CT scans are passed into the DL models, and the ensemble process takes place. The three DL models receive the CT scans as input and generate the feature vectors as output, which are then integrated by the ensemble process. Followed by the SOA is applied to properly tune the hyperparameters involved in the DL models.

#### 3.2.1. RNN Model

In recent times, the RNN technique was extremely preferred, particularly for consecutive data and classic RNN [20]. All nodes at the time step involve input in the preceding nodes, and it remains to use the feedback loops. All the nodes generate the existing hidden form and outcome by employing present input and preceding hidden form as:

(4) $$h_{t}=f\left(W_{h}h_{t-1}+W_{hx}x_{t}+b_{h}\right),\;$$

(5) $$o_{t}=f\left(W_{o}h_{t}+b_{o}\right),\;$$

where $h_{t}$ implies the hidden block of all the time steps (*t*), W represents the weights to the hidden layer from the recurrent link, but b indicates the bias to hidden as well as output forms as f signifies the activation functions executed on all nodes in the networks.

#### 3.2.2. LSTM Model

The major demerit of the conventional RNN technique is that when the time step improves, the network gets failed to derive the context in the time step of the preceding state so much fear after as phenomenon is called long-term dependencies. Because of the deep layer of the network as well as the recurrent performance of classic RNN, explode and vanish gradient issues are also encountered quite frequently. Furthermore, for addressing this issue, the LSTM techniques are established by using memory cells with many gates in hidden layers [20,21]. The block of hidden layers with LSTM cell units and three purposes of gate controller as:

- The forget gate $f_{t}$ chooses that measure of long-term state $c_{t}$ must be omitted;
- An input gate $i_{t}$ control that measure of ${\tilde{c}}_{t}$ must be further to long-term form $c_{t};$
- An output gate $g_{t}$ defines that quantity of $c_{t}$ must be read and output to $h_{t}$ and $o_{t}.$

The subsequent formulas illustrate the long-term as well as short-term forms of cell and output of all layers in time step:

(6) $$f_{t}=\sigma \left(W_{xf}^{T}\cdot x_{t}+W_{hf}^{T}\cdot h_{t-1}+b_{f}\right).\;$$

(7) $$i_{t}=\sigma \left(W_{x}^{T}i\cdot x_{t}+W_{h}^{T}i\cdot h_{t-1}+b_{i}\right).\;$$

(8) $$o_{t}=\sigma \left(W_{x}^{T}o\cdot x_{t}+W_{h}^{T}o\cdot h_{t-1}+b_{i}\right).\;$$

(9) $$g_{t}=tanh\left(W_{x}^{T}g\cdot x_{t}+W_{h}^{T}g\cdot h_{t-1}+b_{i}\right).\;$$

(10) $$c_{t}=f_{t}⊗c_{t-1}+i_{t}⊗{\tilde{c}}_{t}.\;$$

(11) $$o_{t},\;h_{t}=g_{t}⊗tanh\left(c_{t}\right).\;$$

where $W_{x}f,W_{x}i,W_{x}o,W_{x}g$ implies the weight matrices to equivalent linked input vector, $W_{h}f,W_{h}i,W_{h}o,W_{h}g$ defines the weight matrices of the short-term form of preceding time step, and $b_{f},b_{i},b_{o},{\;\mathrm{and}\;b}_{g}\;$ are bias.

#### 3.2.3. GRU Model

In GRU cell units [22], the two vectors in LSTM cells are related as to one vector $o_{t}$. One gate controller controls the combined form of forgetting as well as input gates. If $z_{t}$ output is one, the forget gate was opened, and input gate was closed, but $z_{t}$ was zero, the forget gate was closed, and the input gate was opened. During this case, an input of time step was deleted all the times the earlier (t−1) memory has been saved. During the absence of an output gate, it could be supposed that GRU has various execution of transfer and group of data that LSTM needs for applying. Intuitively, the reset gate defines as combining a novel input with preceding memory, and the upgrade gate chooses the preceding memory data has retained for calculating the novel state. The variances in the outstanding LSTM, but the changes previously defined as:

(12) $$r_{t}=\sigma \left(W_{xr}^{T}\cdot x_{t}+W_{or}^{T}\cdot o_{t-1}+b_{r}\right).\;$$

(13) $$z_{t}=\sigma \left(W_{xz}^{T}\cdot x_{t}+W_{o}^{T}z\cdot o_{t-1}+b_{z}\right).\;$$

(14) $${\tilde{o}}_{t}=tanh\left(W_{x\tilde{o}}^{T}\cdot x_{t}+W_{o\tilde{o}}^{T}\cdot \left(r_{t}⊗o_{t-1}\right)+b_{\tilde{o}}\right).\;$$

(15) $$o_{t}=z_{t}⊗o_{t-1}+\left(1-z_{t}\right)⊗o_{t}.\;$$

where $W_{x}r,\;W_{x}z,\;W_{x\tilde{o}}$ stands for the weight matrices to equivalent linked input vector, $W_{or},\;W_{o}z,\;W_{o}$ implies the weight matrices of preceding time steps, and $b_{r},\;b_{z},\;b_{\tilde{o}}$ are bias.

#### 3.2.4. Ensemble Modeling

The AIEM-DC technique makes use of RNN, LSTM, and GRU models for feature extraction. For aggregating the outcome from these three DL models, they are trained by individual vectors, and 10-fold cross-validation is treated as the fitness function. Consider a data set with a set of k images under x class labels (COVID-19 and non-COVID-19) can be defined by,

(16) $$Imgs=\left\{i_{1},i_{2},\;\ldots ,i_{k}\right\}.\;$$

Assume a set C containing n DL models, in this case, 3 DL models as defined below,

(17) $$C=\left\{\beta_{1},\beta_{2},\;\ldots ,\beta_{n}\right\}$$

The images are fed into the DL model and generate the set *CN*, as expressed in Equation (18):

(18) CN=[β1i1⋯β1ik⋮⋱ βni1 βnik]

Every DL model $\beta_{n}$ offers a decision d∈{−1,1}, related to classification, where 1 denotes non-COVID and −1 for COVID, based on $i_{k}\in \;Imgs$. The decision D can be represented by the use of Equation (19) [21]:

(19) D=[dβ1i1⋯dβ1ik⋮⋱ dβni1 dβnik]

it must be noticed that all elements of matrix D are equal to the outcome of the DNN and image group of CN with respect to place in the matrix, namely $\beta_{n}i_{k}\to d_{\beta_{n}i_{k}}$. Moreover, the score values, s∈{0, …, 1}, has been connected to all the decisions d and demonstrates the posterior probabilities P(ix) which an image i can go to class χ. In addition, the group of scores S is determined as:

(20) S=[P(i1|x)dβ1i1⋯P(ik|x)dβ1ik⋮⋱ P(i1|x)dβni1 P(ik|x)dβnik]. 

During this case, all the elements of matrix S equal to the outcomes of DL techniques and image group of CN with connected posterior probabilities with respect to place in the matrix like $\beta_{n}i_{k}\to d_{\beta_{n}i_{k}}\to P{\left(i_{k}|x\right)}_{d_{\beta_{n}i_{k}}}$.

#### 3.2.5. Hyperparameter Tuning

**Figure 2:**
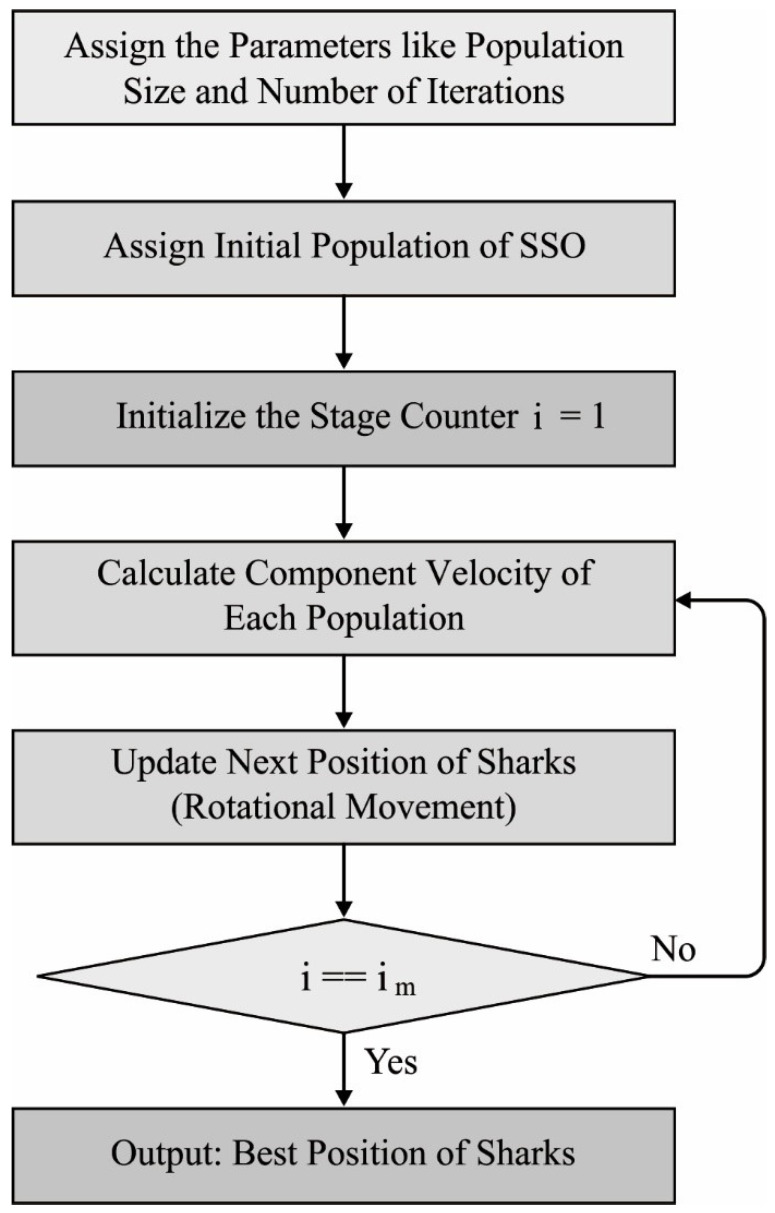
Flowchart of SOA.

In order to tune the hyperparameters (such as batch size, time step, number of layers, learning rate, weight decay, and epoch count) involved in the three DL models, the SOA is employed in such a way that the classification performance gets increased. SOA is an effective bioinspired optimization algorithm [23]. It is commonly employed in different situations [24,25,26], such as cloud job scheduling, resolving arithmetical functions, training ANNs, constructing load forecast, healthcare image development, and optimum process of the reservoir. The SOA was stimulated by the shark behaviors. The rotation motion of sharks is an important operator in the SOA for presenting local optimum. Figure 2 illustrates the flowchart of SOA. In SOA, few assumptions were created, and they are given in the following:

(1) The injured fishes are considered prey to the shark;
(2) The shark tries to discover the injured fish by getting a blood particle from the injured fish’s body;
(3) The velocity of injured fishes is ignored against the shark’s velocity.

In SOA, the shark position is regarded as a candidate solution of the optimization problems [22]:

(21) $$S_{j}^{k}=\left[s_{j,1}^{1},\;s_{j,2}^{1},\;\ldots ,\;s_{j,ND}^{1}\right]$$

where $\;S_{j}^{1}$: the $j^{\mathrm{th}\;}$ primary location, $s_{jk}^{1}$: the $k^{\mathrm{th}\;}$ dimensions of $j^{\mathrm{th}\;}$ sharks’ location, and ND: numbers of d decision variable. While the shark is closer to the injured fish, they obtain stronger odor particles, whereupon they increase their velocity. The shark changes their velocity by:

(22) $$\left|v_{j,\;k}^{i}\right|=min\left[{\left|\eta_{i}\cdot r_{1}\frac{\partial \left(of\right)}{\partial \left(s_{k}\right)}\right|}_{s_{k,\;k}^{i}}+\alpha \cdot r_{2}\cdot v_{j,k}^{i-1}|,\;\left|\beta \cdot v_{j,k}^{i-1}\right|\right]$$

whereas $v_{j,k}^{i}$: the kth dimensions of *j*^th^ sharks velocity, β: velocity limiter, α: inertia coefficient, i: stage numbers, $r_{1},\;r_{2}$: arbitrary number, of the objective function, and $\eta_{i}$: arbitrary numbers. The shark performs the forward movement (*FM*) with the former location and velocity of the shark:

(23) $$P_{j}^{i+1}=S_{j}^{i}+V_{j}^{i}.\Delta t_{i}$$

where $P_{j}^{i+1}$: novel location of *j*^th^ shark according to $FM,\;\Delta t_{i}$: time interval, $S_{j}^{i}$: present place of *j*^th^ sharks, and $\Delta t_{i}$: time interval. The shark uses rotation motion to escape from local optimum:

(24) $$Q_{j}^{i+1,m}=P_{j}^{i+1}+r_{3}.P_{j}^{i+1},\;m=1,\;\ldots ,\;M\;$$

whereas $Q_{j}^{i+1,m}:$ the locations of shark afterward rotation motion, $r_{3}$; arbitrary numbers, M: numbers of point in the local search.

When maximization problems are taken into account, the concluding position of the shark is evaluated by:

(25) $$S_{j}^{i+1}=argmax\left[of\left(P_{j}^{i+1}\right),\;of\left(Q_{j}^{i+1,1}\right),\;\ldots ,\;of\left(Q_{j}^{i+1,M}\right)\right]\;$$

The position of the shark is arbitrarily initiated. Next, the objective functions are calculated for all the agents. The optimal sharks with optimum objective functions are established. Later, the velocity and location of the sharks are upgraded.

### 3.3. Stage 3: IBA-MSVM-Based Classification

At the final stage, the IBA-MSVM model receives the feature vectors as input and allot proper class labels to it. The MSVM classification was dependent upon Vapnik–Chervonenkis (VC) dimensional of the statistical learning system. The key objective of MSVM is to map the preprocessing, non-linear inseparable microarray gene expression information as to a linear extremely dimensional manifold θ with the uses of change $\emptyset :\;R^{N}\to \theta$, afterward attaining an optimum hyperplane: Ψ:ψ(x)=(ω⋅ϕ(x)+b) with resolving the subsequent optimized convex issue (the soft margin issue):

$$\;\min\left(\omega ,\;\xi \right)=\frac{1}{2}\omega^{2}+\beta \overset{n}{\underset{i=1}{\sum }}\xi_{i}$$

Subjected to

(26) $$y_{i}\left(\omega \cdot \phi \left(x\right)+b\right)\geq 1-\xi_{i},\;for\;all\;1\leq i\leq n,\;$$

where ω refers to the coefficient vectors of hyperplane from the manifold (feature space), b implies the threshold value of hyperplanes, $\xi_{i}$ stands for the slack issue presented to classifier error, and β indicates the penalty factor to error [27]. The parameter β controls the penalty of misclassified and their value has been usually defined through cross-validation. Superior values of β generally lead to a small margin that minimizes classifier error, but lower values of β can generate a wider margin resulting from various misclassification.

The feature space θ has been extremely dimensional; hence, their direct calculation leads to “dimensional disaster.” But, as $\omega =\sum_{i=1}^{n}\delta_{i}y_{i}\emptyset \left(x_{i}\right)$, at that point, every operation of MSVM in the feature space θ is only dot products [28]. Then, kernel functions [29], i.e., $\left(x_{i},\;x_{i^{\prime }}\right)=\emptyset \left(x_{i}\right)\cdot \emptyset \left(x_{i^{\prime }}\right)$, are effectual at handle dot product, it can be were presented as to SVM. This represents there is no requirement for knowing to map the microarray gene expression information to their original space to the feature space θ. Therefore, the selection of kernels and their coefficient was essential in the computational performance and accuracy of MSVM classification techniques.

The general kernel function, which is employed as a continuous predictor, contains as:

The linear kernel can be defined as follows.

(27) $$G\left(x_{i},\;x_{i^{\prime }}\right)=x_{i}\cdot x_{i^{\prime }}.\;$$

Next, the polynomial kernel can be represented using Equation (28):

(28) $$G\left(x_{i},\;x_{i^{\prime }}\right)={\left(\eta *\left(x_{i}\cdot x_{i^{\prime }}\right)+\delta \right)}^{d},\;$$

where >0, δ∈R, and $d\in Z^{+}.$

Then, the Gaussian kernel can be equated as follows.

(29) $$G\left(x_{i},\;x_{i^{\prime }}\right)=exp\left(\frac{x_{i}-x_{i}^{2}}{2\sigma^{2}}\right),\;$$

where σ>0.

This MSVM kernel function is approximately considered as follows: local kernel function as well as the global kernel function. Samples widely different have a huge influence on the global kernel values but instanced nearby each other significantly control the local kernel value. The linear, as well as polynomial kernels, were optimum samples of global kernels, but the Gaussian radial basis function (RBF) and Gaussian are local kernels.

Finally, the parameter tuning of the MSVM technique is accomplished by the use of an improved bat algorithm (IBA). BA is a potent optimization method, i.e., broadly employed in distinct applications such as image development domain, parameter extraction of photovoltaic model, satellite formation system, training ELM model, optimum control of power scheme, and FS method [30,31,32]. Excellent and quick convergence in exploitation and exploration are benefits of BA. For getting a sense of distance and finding the variance among food and obstacle, the bat uses their exclusive echolocation capability. In all the iterations, the loudness and pulsation rate of the bats are upgraded. Initially, an arbitrary population of bats is initiated. The bat position is considered a decision variable. The location, frequency, and velocity of the bat changed by:

(30) $$f_{i}=f_{min}+\left(f_{max}-f_{min}\right)\beta \;$$

(31) $$v_{i}^{t}=v_{i}^{t-1}+\left(x_{i}^{t-1}-x^{*}\right)f_{i}$$

(32) $$x_{i}^{t}=x_{i}^{t-1}+v_{i}^{t}$$

whereas β: arbitrary numbers, $f_{min}$: minimal frequency, $f_{max}$: maximal frequency, $x^{*}$: optimal solutions, $v_{i}^{r}$: the velocity of ith bats at iteration $t,\;x_{i}^{t-1}$: the location of ith bats at iteration t−1, $f_{i}$: frequency of ith bats, and $x_{i}^{t}$: the position of ith bats at iteration t. The bat uses an arbitrary walk as a local search:

(33) $$x_{new}=x_{old}+\varepsilon A^{t}$$

$x_{new}$: novel location of bats, $x_{old}$: old location of bats, $A^{t}$: loudness, and ε: arbitrary numbers. The bat’s loudness and pulsation rate are different by:

(34) $$A_{i}^{t+1}=\vartheta A_{i}^{t},r_{i}^{t+1}=\left[1-exp\left(-\gamma^{t}\;\right)\right]$$

In which ϑ and γ: constants, $r_{i}^{t+1}$: pulsation rate of *i*th bats, $A_{i}^{t+1}$: loudness of ith bats at iteration t+1. Initially, the first population and arbitrary value of the parameters are determined [24]. Next, the value of objective functions is calculated for all bats to define the quality of the solution. Lastly, the optimal bat with optimal values of the objective functions are determined, the velocity and position of bats are upgraded.

The IBA technique is derived by the use of Lévy flight (HH). This process was employed for more relieving the premature convergence problem that is the core drawback of BA. The Lévy flight (LF) [33] offers an arbitrary walk process for prospering management of local search. This procedure was demonstrated as:

(35) $$Le\;\left(w\right)\approx w^{-1-\tau }$$

(36) $$w=\frac{A}{|B{|}^{\frac{1}{\tau }}\;}$$

(37) $$\sigma^{2}={\left\{\frac{\Gamma \left(1+\tau \right)}{\tau \Gamma \left(\frac{1+\tau }{2}\right)}\frac{\sin\left(\frac{\pi \tau }{2}\right)}{2^{\frac{1+\tau }{2}}}\right\}}^{\frac{2}{\tau }}$$

where $0<\tau \leq 2,\;A∼N\left(O,\;\sigma^{2}\right)$ and $B∼N\left(O,\;\sigma^{2}\right),\;\Gamma \left(.\right)$ implies the Gamma function, w explains the step size, τ stands for the Lévy index, A/B∼N(O, σ2) implies that instances create from Gaussian distribution in that mean is 0 and variance is $\sigma^{2}$ correspondingly. According to the above-mentioned process, a novel enhanced part to upgrade the solutions of BA as:

(38) $${\vec{D}}_{el}={\vec{D}}_{e}+\left|{\vec{P}}_{N}+{\vec{d}}_{e}\right|\times Le\left(\delta \right)$$

where $D_{e}|$ signifies the novel place of search agents $D_{e}.$ To guarantee the optimum solution candidate, a fitter agent is kept:

(39) $$D_{e}|=\{\begin{array}{l} D_{e}|\;F(D_{e}|)>F\left(D_{e}\right)\\ D_{e}\;otherwise \end{array}$$

## 4. Experimental Validation

### 4.1. Data Set Details

**Figure 3:**
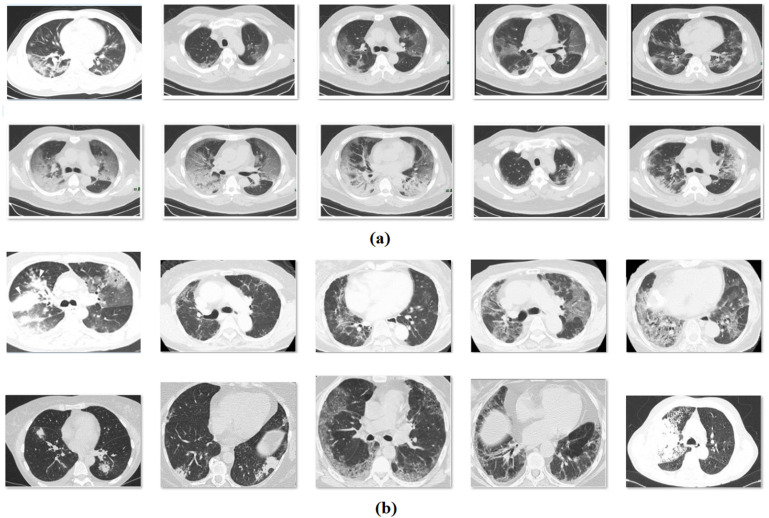
(**a**) COVID, (**b**) non-COVID: sample images.

This section assesses the performance of the proposed model on the benchmark COVID-CT-data set [34], which includes 349 CT images with the clinical findings of COVID-19 from 216 patients. The images are collected from COVID-19-related papers from medRxiv, bioRxiv, NEJM, JAMA, Lancet, etc. CTs containing COVID-19 abnormalities are selected by reading the figure captions in the papers. Figure 3 depicts the sample test images. Moreover, we have used 10-fold cross-validation to split the data set into training and testing parts.

### 4.2. Results and Discussion

**Figure 4:**
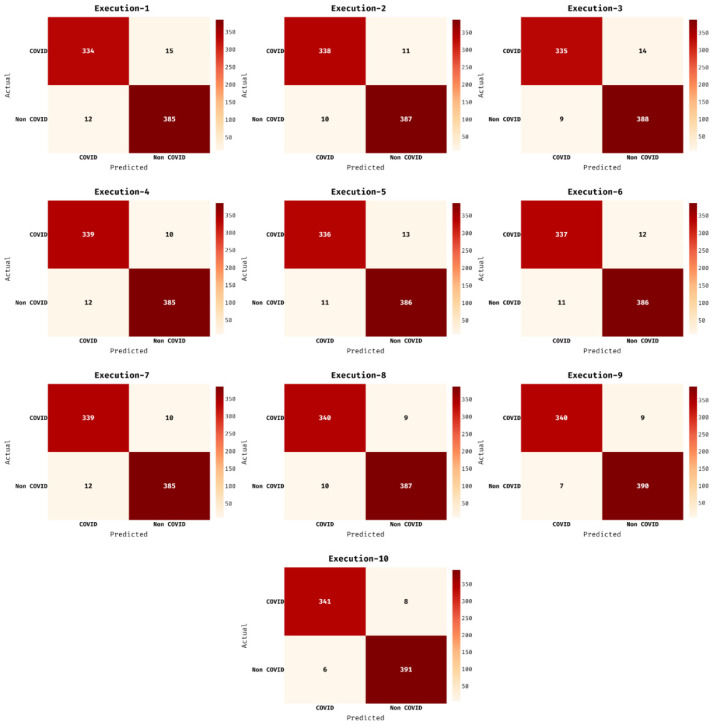
Confusion matrix of AIEM-DC technique under ten executions.

Figure 4 demonstrates the confusion matrices of the AIEM-DC techniques under 10 executions. The results exhibited that the AIEM-DC technique has demonstrated effectual outcomes under every execution. For instance, with execution-2, the AIEM-DC technique has classified a set of 338 images into COVID and 391 images into non-COVID. Followed by, with execution-4, the AIEM-DC method has ordered a set of 339 images into COVID and 385 images into non-COVID. In line with, with execution-6, the AIEM-DC approach has classified a set of 337 images into COVID and 386 images into non-COVID. Moreover, with execution-8, the AIEM-DC manner has categorized a set of 340 images into COVID and 387 images into non-COVID. At last, with execution-10, the AIEM-DC algorithm has classified a set of 341 images into COVID and 391 images into non-COVID.

**Figure 5:**
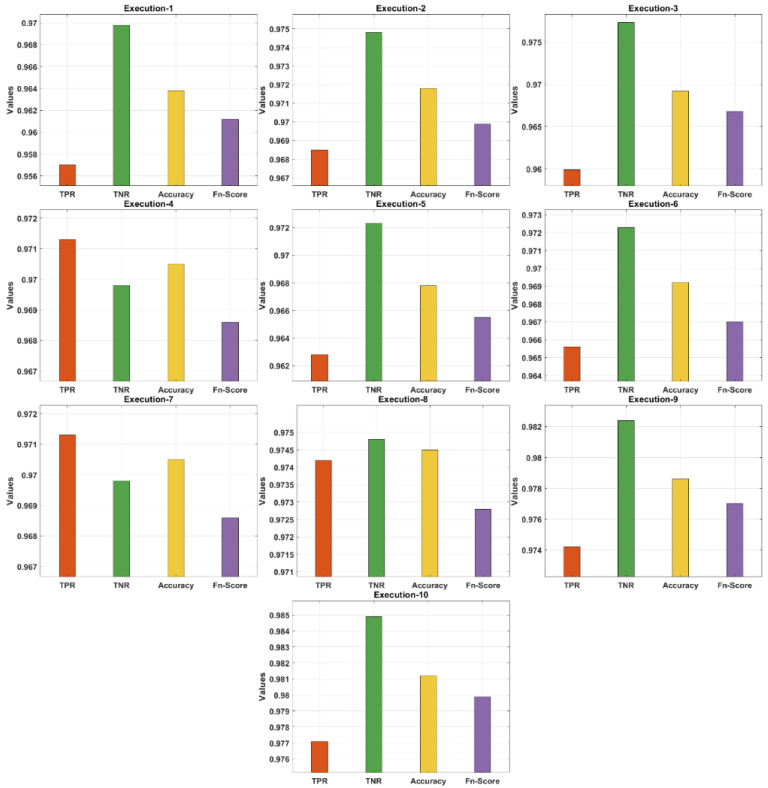
Result analysis of AIEM-DC model with different measures.

Table 1 and Figure 5 demonstrate the confusion matrix of the AIEM-DC technique under 10 execution rounds. The obtained results highlighted the effectual outcome of the AIEM-DC technique under every round. For instance, with execution-1, the AIEM-DC technique has obtained a TPR of 0.9570, TNR of 0.9698, accuracy of 0.9638, and F-score of 0.9612. In addition, with execution-4, the AIEM-DC method has gained a TPR of 0.9713, TNR of 0.9698, accuracy of 0.9705, and F-score of 0.9686. In addition, with execution-6, the AIEM-DC approach has reached a TPR of 0.9656, TNR of 0.9723, accuracy of 0.9692, and F-score of 0.9670. Moreover, with execution-8, the AIEM-DC system has achieved a TPR of 0.9742, TNR of 0.9748, accuracy of 0.9745, and F-score of 0.9728. Finally, with execution-10, the AIEM-DC methodology has gained a TPR of 0.9771, TNR of 0.9849, accuracy of 0.9812, and F-score of 0.9799.

**Figure 6:**
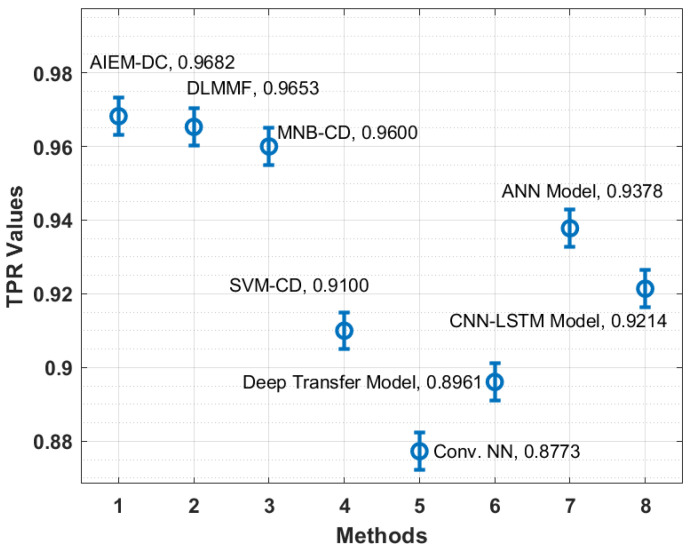
TPR analysis of AIEM-DC model with existing approaches.

In order to showcase the effectual outcome of the AIEM-DC technique, a detailed comparison study is made with recent techniques in Table 2 [35]. Figure 6 showcases the TPR analysis of the AIEM-DC technique with existing techniques. The figure demonstrated that the Conv. NN and deep transfer models have obtained ineffective outcomes with the lower TPR of 0.8773 and 0.8961, respectively. In addition, the SVM-CD and CNN-LSTM techniques have attained slightly enhanced TPR of 0.9100 and 0.9214, respectively. Followed by, the ANN and MNB-CD techniques have showcased reasonable TPR of 0.9378 and 0.9600, respectively. Furthermore, the DLMMF technique has accomplished near-optimal outcomes with the TPR of 0.9653. However, the proposed technique has resulted in improved performance with a TPR of 0.9682.

**Figure 7:**
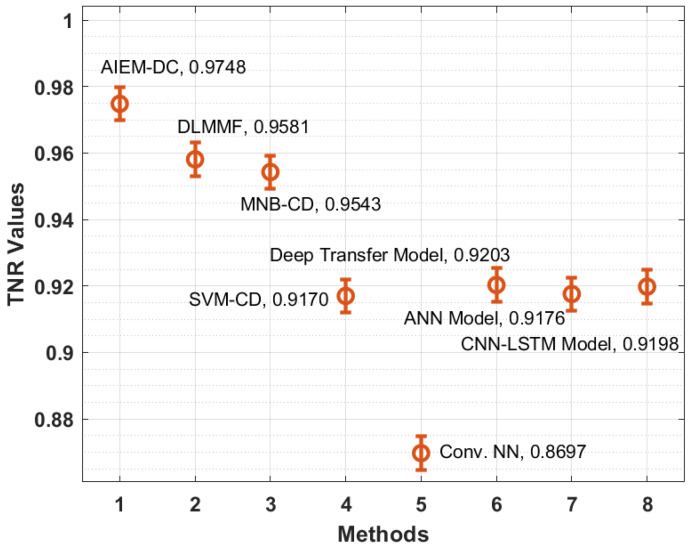
TNR analysis of the AIEM-DC model with existing approaches.

Figure 7 illustrates the TNR analysis of the AIEM-DC approach with recent methods. The figure depicted that the Conv. NN and SVM-CD manners have attained ineffective outcomes with the minimal TNR of 0.8697 and 0.9170 correspondingly. Moreover, the ANN and CNN-LSTM methods have reached somewhat improved TNR of 0.9176 and 0.9198 correspondingly. In addition, the deep transfer and MNB-CD techniques have demonstrated a reasonable TNR of 0.9203 and 0.9543 correspondingly. Moreover, the DLMMF manner has accomplished near optimum outcomes with the TNR of 0.9581. However, the proposed methodology has resulted in increased performance with the TNR of 0.9748.

**Figure 8:**
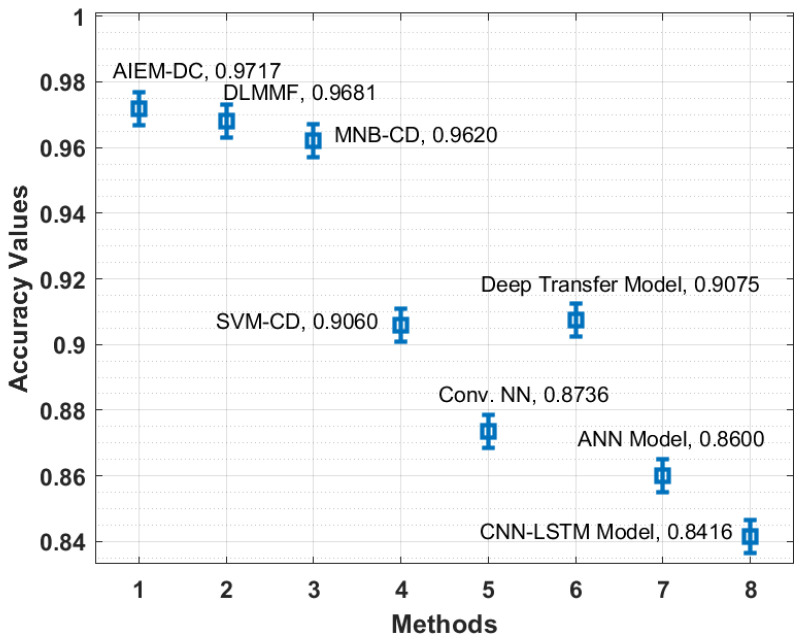
Accuracy analysis of the AIEM-DC model with existing approaches.

Figure 8 depicts the accuracy analysis of the AIEM-DC method with state-of-the-art algorithms. The figure outperformed that the CNN-LSTM and ANN manners have gained ineffective outcomes with the least accuracy of 0.8416 and 0.8600 correspondingly. Moreover, the Conv. NN and SVM-CD manners have achieved slightly superior accuracy of 0.8736 and 0.9060, respectively. At the same time, the deep transfer and MNB-CD techniques have depicted reasonable accuracy of 0.9075 and 0.9620 correspondingly. Additionally, the DLMMF algorithm has accomplished near-optimal results with an accuracy of 0.9681. Finally, the presented technique has resulted in maximum efficiency with an accuracy of 0.9717.

**Figure 9:**
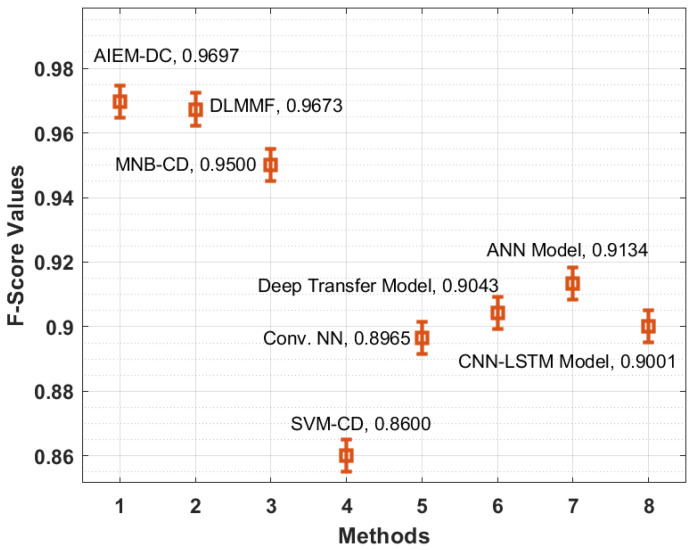
F-score analysis of the AIEM-DC model with existing approaches.

Figure 9 demonstrates the F-score analysis of the AIEM-DC manner with existing methods. The figure stated that the SVM-CD and Conv. NN methodologies have gained ineffective outcomes with the minimum F-score of 0.8600 and 0.8965 correspondingly. In addition, the CNN-LSTM and deep transfer techniques have reached somewhat increased F-score of 0.9001 and 0.9043 correspondingly. Similarly, the ANN and MNB-CD approaches have outperformed reasonable F-score of 0.9134 and 0.9500 correspondingly. Moreover, the DLMMF approach has accomplished near-optimal outcomes with an F-score of 0.9673. At last, the proposed method has resulted in maximal performance with an F-score of 0.9697.

## 5. Conclusions

In this study, a new AIEM-DC technique is proposed for the detection and classification of COVID-19 using chest CT scans. The AIEM-DC technique aims to accurately detect and classify the COVID-19 using an ensemble of DL models. The AIEM-DC technique involves GF-based preprocessing, ensemble DL-based feature extraction, SOA-based hyperparameter tuning, MSVM-based classification, and IBA-based parameter tuning. The design of SOA and IBA techniques paves a way to improve the overall classification performance of the AIEM-DC technique to a maximum extent. The experimental validation of the AIEM-DC technique is validated on the benchmark CT image data set, and the results reported the promising classification performance of the AIEM-DC technique over the recent state-of-the-art approaches. As a part of future extension, the hybrid DL architectures can be designed to boost the classification performance of the AIEM-DC technique.

## Figures and Tables

**Table 1:** Results analysis of proposed AIEM-DC model in terms of various measures.

No. of Execution	TPR	TNR	Accuracy	F-Score
Execution-1	0.9570	0.9698	0.9638	0.9612
Execution-2	0.9685	0.9748	0.9718	0.9699
Execution-3	0.9599	0.9773	0.9692	0.9668
Execution-4	0.9713	0.9698	0.9705	0.9686
Execution-5	0.9628	0.9723	0.9678	0.9655
Execution-6	0.9656	0.9723	0.9692	0.9670
Execution-7	0.9713	0.9698	0.9705	0.9686
Execution-8	0.9742	0.9748	0.9745	0.9728
Execution-9	0.9742	0.9824	0.9786	0.9770
Execution-10	0.9771	0.9849	0.9812	0.9799
Average	0.9682	0.9748	0.9717	0.9697

**Table 2:** Comparative analysis of existing with proposed AIEM-DC method with recent methods [35].

Methods	TPR	TNR	Accuracy	F-Score
AIEM-DC (Ours)	0.9682	0.9748	0.9717	0.9697
DLMMF	0.9653	0.9581	0.9681	0.9673
MNB-CD	0.9600	0.9543	0.9620	0.9500
SVM-CD	0.9100	0.9170	0.9060	0.8600
Conv. NN	0.8773	0.8697	0.8736	0.8965
Deep Transfer Model	0.8961	0.9203	0.9075	0.9043
ANN Model	0.9378	0.9176	0.8600	0.9134
CNN-LSTM Model	0.9214	0.9198	0.8416	0.9001

## Data Availability

Data sharing does not apply to this article as no data sets were generated during the current study.
